# Research on Rapid Detection Technology for *β*_2_-Agonists: Multi-Residue Fluorescence Immunochromatography Based on Dimeric Artificial Antigen

**DOI:** 10.3390/foods11060863

**Published:** 2022-03-18

**Authors:** Miaomiao Liu, Biao Ma, Yaping Wang, Erjing Chen, Jiali Li, Mingzhou Zhang

**Affiliations:** Zhejiang Provincial Key Laboratory of Biometrology and Inspection & Quarantine, China Jiliang University, Hangzhou 310018, China; miaoliull@163.com (M.L.); 16a0701109@cjlu.edu.cn (B.M.); wyping524@163.com (Y.W.); s1709071002@cjlu.edu.cn (E.C.); qjc1993@126.com (J.L.)

**Keywords:** *β*_2_-agonists, dimeric artificial antigen, fluorescent lateral flow immunoassay, europium nanoparticles, multi-residue analysis

## Abstract

To detect two types of *β*_2_-agonist residues at the same time, we coupled two haptens of clenbuterol (CLE) and ractopamine (RAC) to the same carrier protein through diazotization to prepare dimeric artificial antigen, and a fluorescent lateral flow immunoassay method based on europium nanoparticles (EuNP-FLFIA) was established by combining polyclonal antibodies with europium nanoparticles to form probes. Under optimized conditions, the EuNP-FLFIA could simultaneously detect eight aniline-type and one phenol-type *β*_2_-agonists, and the limits of detection (LOD) were 0.11–0.19 ng/mL and 0.12 ng/mL, respectively. The recovery rate of this method was 84.00–114.00%. This method was verified by liquid chromatography–tandem mass spectrometry (LC-MS/MS), and the test results were consistent (R^2^ > 0.98). Therefore, the method established in this study could be used as a high-throughput screening for the efficient and sensitive detection of *β*_2_-agonists in food.

## 1. Introduction

*β*_2_-agonists have the effects of promoting protein synthesis, enhancing muscle growth, and reducing fat tissue deposition, so they can be applied as growth promoters [1]. Hence, they have often been abused as growth promoters for cattle, pigs, and other farm animals [2,3]. Due to the adverse effects of these drug residues on humans, such as food poisoning, cardiovascular diseases, and central nervous system diseases [4], the use of these drugs was prohibited in many countries [5,6], so it was particularly significant to establish a rapid and highly sensitive method for the detection of *β*_2_-agonist residues.

In recent decades, a variety of analytical methods for detecting *β*_2_-agonists has been developed, such as high-performance liquid chromatography (HPLC) [7], gas chromatography–mass spectrometry (GC-MS) [8], liquid chromatography–mass spectrometry (LC-MS) [9], liquid chromatography–tandem mass spectrometry (LC-MS/MS) [10], capillary electrophoresis electrochemical detection [11], colorimetric ELISA [12], and other analytical methods. Although large instrumental methods can accurately detect complex samples, they often require time-consuming procedures, expensive instruments, and skilled technicians and are not suitable for rapid screening and onsite analyses [13]. As an alternative, a lateral flow immunoassay has unique advantages, such as being fast, easy-to-use, portable, and low-cost, and has been promoted to detect veterinary drug residues [14]. The most commonly used form of lateral flow immunoassay is to use colloidal gold as the reporting medium for colorimetric detection, which can achieve a qualitative [15] or semi-quantitative analysis of the target analytes [16]. However, this form of lateral flow immunoassay can only be used to analyze target analytes with relatively high concentrations [17]. Fluorescent nanoparticles can enhance the detection signal and improve the detection sensitivity when used in lateral flow immunoassays, so they have received extensive attention. The development of fluorescent signals has attracted widespread attention, because it can enhance the detection signal and improve the sensitivity of a lateral flow immunoassay [15,18]. Europium nanoparticles (EuNPs), also known as Eu (III)-labeled polystyrene nanoparticles, are a new type of fluorescent probe based on lanthanide labeling [19]. EuNPs are special functional microspheres, and each microsphere contains thousands of fluorescent molecules [20]. They have the advantages of good stability, high labeling efficiency, and high sensitivity [21], and they are not harmful to the sample [22]. Therefore, EuNPs have been used in food safety analysis [23] and medical diagnosis [24]. For example, compared with a colloidal gold rapid diagnostic kit, the sensitivity of a fluorescent lateral flow immunoassay method based on europium nanoparticles (EuNP-FLFIA) using EuNPs was significantly improved [25]. Hence, EuNPs as markers could provide a good tool for improving the sensitivity of the fluorescent lateral flow immunoassay detection.

The *β*_2_-agonists included phenol types and aniline types [26]. Both types need to be detected when detecting multiple veterinary drug residues in pig products. In the existing detection methods, artificial antigens are mostly prepared for one type of *β*_2_-agonist, and the detection target is limited to a specific type. In recent years, broad-spectrum antibodies with multiple recognition properties have attracted great interest. Compared with a single analyte immunoassay, a multi-analyte immunoassay has some significant advantages, such as high sample throughput, improved analysis efficiency, low sample consumption, and a lower total cost per analysis [27,28]. At present, the common and effective method for a broad-spectrum immunoassay is to generate broad-spectrum antibodies by using universal hapten [29]. However, this immunoassay still had no extensive specificity for *β*_2_-agonists with different structures [30,31].

Clenbuterol (CLE) belongs to the aniline group of *β*_2_-agonists [32], and ractopamine (RAC) belongs to the phenol group of *β*_2_-agonists [33]. In this study, a CLE-RAC dimer artificial antigen was synthesized by the diazotization reaction of CLE and RAC with a carrier protein to prepare an anti-CLE-RAC polyclonal antibody, which achieved the effect of simultaneously determining two major *β*_2_-agonists of a phenol type and aniline type in one experiment. A novel, broad-spectrum immunoassay based on EuNP-FLFIA for the simultaneous detection of aniline and phenol *β*_2_-agonists was developed. This strategy was effective and allowed for a broader selection of *β*_2_-agonists.

## 2. Materials and Methods

### 2.1. Reagents and Chemicals

CLE, RAC, phenylethanolamine A (PhA), Salbutamol (SAL), Cloprenaline (CLO), Cimbuterol (CIMB), Bromobuterol (BRO), Tulobuterol (TUL), Mapenterol (MAP), Cimaterol (CIM), Mabuterol (MAB), Zilpaterol (ZIL), Bambuterol (BAM), and Clenproperol (CLEN) standards were purchased from the National Institute of Metrology, P.R. China (Beijing, China). Bovine Serum Albumin (BSA), human serum albumin (HSA), sodium nitrite, sulfuric acid, starch potassium iodide test paper, labeled goat anti-rabbit IgG, Tween-20, and glycerol were purchased from Sigma-Aldrich (St. Louis, MO, USA). 2-(*N*-Morpholino) etha-nesulfonic acid (MES) was purchased from Yuchun Biological Technology Co., Ltd. (Shanghai, China). 1-(3-Di-methylaminopropyl)-3-ethylcarbodiimide hydrochloride (EDC) was supplied by Hengdai Lao Biological Co., Ltd. (Shanghai, China). Carboxylate-modified EuNPs (200 nm in diameter) were purchased from Shanghai Uni Biotech Ltd. (Shanghai, China). Tetramethylbenzidine (TMB) was purchased from Boyao Biological Technology Co., Ltd. (Shanghai, China). Nitrocellulose (NC) membrane, sample pad, conjugate pad, backing card, and absorbent pad were obtained from Dean Biotechnology Co., Ltd. (Hangzhou, Zhejiang, China). Other conventional chemical reagents were purchased from Sinopharm Group Chemical Reagent Co., Ltd. (Shanghai, China).

We weighed 8 g of NaCl, 0.2 g of KCl, 1.44 g of Na_2_HPO_4_, and 0.24 g of KH_2_PO_4_; dissolved them in 800 mL of distilled water; adjusted the pH of the solution to 7.4 with HCl; and diluted it to 1 L with distilled water to obtain a phosphate-buffered saline (PBS, 0.01 M, pH 7.4). A borate buffer solution (BBS, 0.05 m, pH 8.2) was prepared by dissolving 0.81 g of boric acid and 0.67 g of trisodium tetroxide in 1 L of ultra-pure water and adjusting the pH to 8.2. We weighed 3.18 g of Na_2_CO_3_ and 5.88 g of NaHCO_3_ in ultra-pure water to a final volume of 2 L to produce a carbonate buffered solution (CBS, 0.1 M, pH 9.6).

### 2.2. Apparatus

The XYZ3000 dispensing platform was used for scribing, and the CM2000 guillotine cutter was used for cutting test strips (BioDot, Irvine, CA, USA). A F-4500 fluorescence spectrometer (Hitachi, Tokyo, Japan) was used to detect the test strip data. The colloidal gold test strip results were read using a GY-610 colloidal gold test strip reader (Henan Guanyu Instrument Co., Ltd., Zhengzhou, Henan, China). The FIC-S2011 dry fluorescence immunoassay analyzer (Suzhou Helmen Precise Instruments, Suzhou, Jiangsu, China) was used to read the fluorescent strip data.

### 2.3. Synthesis of Dimeric Artificial Antigen

In this study, p-hydroxybenzaldehyde and nitromethane were used as starting materials, and the Raney nickel reduction method was used to synthesize RAC-NH_2_ derivatives. A total of 53.0-mg CLE and 55.2-mg RAC-NH_2_ derivative were weighed in the reaction system with a balance. We added 6.2 mL of 0.5-mol/L sulfuric acid in a 37 °C water bath for 20 min, allowed it to dissolve completely, precooled it at 4 °C, and added 115.4 µL of 10-mg/mL sodium nitrite solution over ice with stirring. It was reacted at 4 °C for 3 h while stirring. After the reaction was completed, the excess NaNO_2_ was removed by ammonium sulfamate. Dropwise, 10 mL of HSA solution (125 mg of HSA in 10 mL of 1-mol/L carbonate buffer) was added to the reaction system conducted above. We maintained the pH of the reaction environment at 9.0 with 1-mol/L NaOH solution for 4 h while stirring. Finally, the fully reacted reaction solution was dialyzed for 4 consecutive days, and the solution was changed twice on day 1. The dialyzed solution was stored at −20 °C for subsequent use. One of the CLE-HSA-RAC with the best synthetic effect was selected as the immunogen to immunize the rabbit to produce an antibody, and then, the next work, polyclonal antibody preparation was performed. CLE-BSA-RAC was used as the coating antigen, and the synthesis method was similar to the above (The schematic diagram is shown in Figure S1).

### 2.4. Preparation of Polyclonal Antibodies

In experiments that require the use of animals, this experiment always followed the “Zhejiang Provincial Laboratory Animal Management Method”. Two adult New Zealand rabbits were immunized with CLE-HSA-RAC. Each rabbit was injected with 1 mg of immunogen, which was dissolved in 0.5 mL of normal saline and emulsified with the same volume of Freund’s complete adjuvant. Intradermal injection was performed at four sites on the back of the animal. For boosted immunity, an equal volume of incomplete Freund’s adjuvant was used. Four follow-up injections were given at four-week intervals. Before each injection, blood was drawn from the marginal vein of the rabbit’s ear and adjusted to 2000× *g* and centrifuged at 4 °C for 10 min, and the serum was assayed for the antibody titer by ELISA. Animals were exsanguinated 10 days after the last immunization. The serum was separated, and the antibody was extracted by the octanoic acid–ammonium sulfate method [34], subpackaged, and stored at −20 °C. The titer of the antibody detected by indirect competitive ELISA was determined to be positive if the OD_450_ nm/negative OD_450_ nm of the well tested ≥2.1 (P/N ≥ 2.1).

### 2.5. Preparation of EuNP Polyclonal Antibody Probes

This combination process was based on our previous work [20]. We added 1 mg of carboxylic acid EuNPs to a 400-μL MES solution and then added 15-μL EDC (10 mg/mL) to activate after slowly shaking for 30 min at room temperature (RT). After centrifuging the activation solution, the excess EDC was separated, and the precipitate was dissolved in 500-μL BBS (0.05 M, pH 8.2) by a supersonic wave. Then, we added 1 mL of polyclonal antibody solution at 2.5, 5, 10, 20, and 40 μg/mL and mixed it gently under RT for 2 h. After the reaction, 55 μL of blocking buffer and 10% BSA (*w/v*) were added, and EuNP polyclonal antibody conjugates were centrifuged at 13,000 rpm twice to discard the unreacted antibodies and BSA. Finally, the sediment was resuspended in a 500-μL preservation solution containing 0.1% BSA (*w/v*) (0.05 M, pH 8.2) and stored at 4 °C for standby.

### 2.6. Preparation of Colloidal Gold Polyclonal Antibody Probes

According to the sodium citrate reduction method used in the previous experiment [35], we prepared colloidal gold (CG) nanoparticles with chloroauric acid in the presence of a reducing agent of sodium citrate and conjugated with polyclonal antibody.

### 2.7. Preparation of Lateral Flow Strips

The immunochromatographic test strip was composed of a sample pad, conjugate pad, nitrocellulose membrane, absorbent pad, and backing card. The sample pads and conjugate pads were made of the same specification of fiberglass. We soaked them in a PBS buffer (0.01 M, pH 7.4) containing 1%BSA (*w/v*) and 0.05% Tween-20 (*v/v*) for 30 min, then put them in a dryer at 37 °C for 2 h and stored them in a sealed bag with desiccant under RT for use. The test line (T line) was at the bottom of the membrane, on which CLE-BSA-RAC sprayed goat anti-rabbit IgG to the top of the membrane as the control line (C line) of the test strip (the distance between the C line and T line was 70 mm). The labeled polyclonal antibody was sprayed onto the conjugate pad and dried at 37 °C overnight. The NC membrane was manually attached to the backing card, and the bonding pad was affixed to the bottom of the NC membrane, which had a 2-mm overlap with NC membrane. The sample pad was affixed to the bottom of the binding pad in the same way, and the water absorption pad was attached to the top of the NC membrane, which overlapped the NC membrane by 2 mm. Then, the entire assembled long strip was divided into 2.5-mm-wide strips and dried and stored at RT for later use.

### 2.8. EuNP-FLFIA Detection Procedure

When using the immunochromatographic test strip to analyze the sample, for EuNP-FLFIA, we took out 50 μL of standard solution or sample solution and placed it on the sample pad and observed the results under a portable UV lamp at 365 nm; the data were read within the optimum reaction time. For colloidal gold-lateral flow immunoassay (CG-LFIA), we took 50 μL of the test solution and dropped it on the sample pad and directly observed the result after a period of time. At the same time, we put the test strip into the test strip reader and recorded the results of the T and C lines.

### 2.9. Parameter Optimization

In order to make immunoassays with higher sensitivity and faster detection, it is crucial to select and optimize a suitable detection system. The effects of certain parameters on the performance of the system were investigated—in particular, the immune reaction time, the properties of the buffer, the concentration of labeled antibody, the material of the nitrocellulose membrane, the concentration of the EuNP polyclonal antibody probe, the concentration of the artificial antigen, and the concentration of goat anti-rabbit IgG.

The fluorescence intensities of the C and T lines of the test strips were measured at 5, 10, 15, and 20 min after the start of the reaction, respectively, and the optimal reaction time was selected by comparison.

Four different buffers were tested, namely ultra-pure water (pH 5.0), BBS buffer (0.05 m, pH 8.2), PBS buffer (0.05 m, pH 7.4), and CBS buffer (0.05 m, pH 9.6). The effects of the solutions and their pH on the sensitivity of the assay were tested and optimized, respectively.

A total of 10 μL of europium microsphere solution was reacted with 2.5, 5, 10, 20, and 40 μg/mL of polyclonal antibody, from which the optimal amount of antibody labeling was selected based on the fluorescence intensity.

The fluorescence intensities of the EuNP polyclonal antibody probes in the concentration range of 1–8 ng/mL were measured individually to select the optimal concentration.

The concentrations of CLE-BSA-RAC and goat anti-rabbit IgG were optimized from a range of 0.4, 0.6, 0.8, 1.0, and 1.2 mg/mL and 0.25, 0.5, 1, and 2 mg/mL, respectively. The materials of the nitrocellulose membranes were also selected. Six different types of nitrocellulose membranes were selected, and the best of them was chosen for the subsequent experiments based on fluorescence intensity.

### 2.10. Sensitivity and Specificity

To determine the sensitivity of EuNPs-FLFIA, the standards of RAC and CLE were mixed at the same concentration, and it diluted to the final concentrations of 0.05, 0.1, 0.25, 0.5, 1, 2.5, 5, and 10 ng/mL and diluted with BBS buffer (0.05 M, pH 8.0); a competition curve was obtained by plotting the intensity of inhibition against the logarithm of the analyte concentration under optimized conditions. The 50% inhibition value (IC_50_) and limit of detection (IC_10_, LOD) were obtained from a four-parameter logistic equation of the sigmoidal curve [36].

The IC_50_ values of CLE, RAC, PEAA, SAL, CLO, CIMB, BRO, TUL, MAP, CIM, MAB, ZIL, BAM, TER, and CLEN were respectively detected by the method, and the specificity was calculated. The specificity of the immunochromatography was evaluated by measuring the cross-reactivity (CR) of a group of structurally related analytes. The CR values were calculated using this formula: CR (%) = (IC_50_ of CLE/IC_50_ of analyte) × 100.

### 2.11. Analysis of Spiked Recoveries

To assess the accuracy of the developed test strips, we prepared spiked samples at concentrations of 0.5–2 ng/mL and tested by the EuNP-FLFIA method under optimized conditions by adding *β*_2_-agonists standards to blank negative swine urine.

### 2.12. Analysis of Actual Samples

To further validate the developed EuNP-FLFIA analytical method, 60 swine urine, serum, muscle, and liver samples from Zhejiang Province were analyzed using both the EuNP-FLFIA analytical method and the LC-MS/MS method. Among them, since not all *β*_2_-agonists were recognized by the polyclonal antibody obtained, 9 kinds of *β*_2_-agonists, which were detected by EuNP-FLFIA, were tested by LC-MS/MS, and the others were not for comparison.

The sample pretreatment method was similar to that of Li et al. [37]. For serum and urine samples, 6 mL of 50-mM ammonium acetate solution (pH 5.2) was added to 2 mL of urine or serum to extract the samples. Then, 40 μL of *β*-glucuronidase was added, and the mixture was vortexed in a water bath at 37 °C for 16 h. After cooling to room temperature, 2 mL of 6% perchloric acid was added to precipitate protein in the serum samples. Then, the sample solution was processed by freezing centrifugation at 7012× *g* at 4 °C for 10 min. We removed 2 mL of the solution and dried it in a 60 °C water bath under nitrogen flow, then reconstituted it in 400 μL of PBS for use. For muscle and liver, before extraction, the tissue was ground in a chopper for five minutes. Then, 2 g of tissue fragments and 6 mL of 50-mM ammonium acetate solution (pH 5.2) were added into a 50-mL polypropylene centrifuge tube, and the tissue was homogenized for 3 min. The next steps were similar to those in the serum sample analysis.

Actual samples were tested for residual *β*_2_-agonists by LC-MS/MS according to the national standards. The chromatographic column was an Acquity BEH C_18_ column (100 mm × 2.1 mm, 1.7 μm), and the mass spectrometry detection was performed on a Micromass Quattro Premier XE system (Waters, Manchester, UK) equipped with an electrospray ionization (ESI) source. The detection experiment selected the positive mode and multiple reaction monitoring. The parameters were set to source temperature, 120 °C; capillary voltage, 3000 V; desolventizing temperature, 300 °C; cone gas (N_2_) flow rate, 60 L/h; desolventizing gas (N_2_) flow rate, 750 L/h; and collision cell pressure, 4 × 103 mbar. All samples were analyzed by EuNP-FLFIA and confirmed by LC-MS/MS measurement.

### 2.13. Data Analysis

The fluorescence spectra required in the text were analyzed using Origin 9.0 software (Origin Lab, Northampton, MA, USA). Data were plotted into visual formats such as graphs for analysis using Microsoft Excel software (Excel 2010, Microsoft Corporation, Redmond, WA, USA). Photoshop software (Photoshop CS3, Adobe Systems, San Jose, CA, USA) was used to draw the schematic diagram of the test strip.

## 3. Results and Discussion

### 3.1. Detection Principle

**Figure 1:**
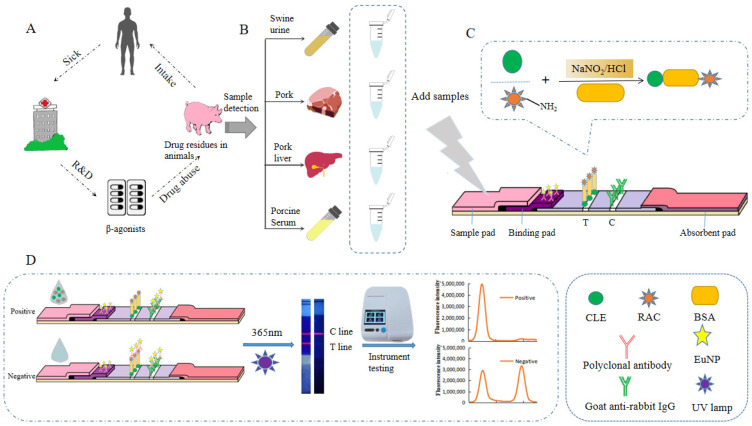
The EuNP-FLFIA detection principle. (**A**) The effects of *β*_2_-agonist abuse on the human body through the food chain. (**B**) Detection of actual samples. (**C**) Synthesis of dimeric artificial antigen. (**D**) The testing process and test results of the EuNP-FLFIA reader test results.

The schematic diagram of test strip detection is shown in Figure 1. The detection principle was based on the target analyte competing with the dimeric artificial antigen immobilized on the nitrocellulose membrane to bind to the EuNP-labeled polyclonal antibody. CLE-BSA-RAC was coated on a nitrocellulose membrane as a capture reagent. The EuNP-labeled polyclonal antibody was fixed on the binding pad as the detection probe. We dropped the sample solution containing the target analyte into the sample pad, and it flowed through the capillary to the other side. If the sample contained RAC or CLE, RAC or CLE would bind to the EuNP polyclonal antibody probes, and the T line would not show color. Excess EuNP probes would continue to diffuse upward and be captured by goat anti-rabbit IgG on the C line and develop color. If the sample did not contain RAC or CLE, EuNP probes would be intercepted by RAC-BSA-CLE, and the T lines would be stained. Regardless of the presence of RAC or CLE in the sample, when the EuNP probes diffused to the C line, goat anti-rabbit IgG would capture the EuNP probes, and the C line would color. After the reaction, the fluorescence signal intensity of the test paper was read by a fluorescence reader and stored.

### 3.2. Identification of the CLE-RAC Dimeric Artificial Antigen and Polyclonal Antibodies

The prepared CLE-BSA-RAC and CLE-HSA-RAC were identified by SDS-PAGE electrophoresis and the UV–Vis spectrum (Supplementary Materials Figures S2 and S3).

The titer determination results obtained by indirect ELISA showed that the titers of the antisera produced by the two immunized New Zealand rabbits were both more than 1:128,000, indicating that the injected immunogen had a good immune effect (Supplementary Materials Table S1).

### 3.3. Optimization of the EuNP-FLFIA Parameter

To obtain more rapid and highly sensitive results, the immune response time, composition of the buffers, the concentration of the labeled antibody, different NC membrane options, the concentration of CLE-BSA-RAC, and the influence of the concentration of EuNP polyclonal antibody probes on the fluorescence intensity will all have a certain effect on the result.

**Figure 2:**
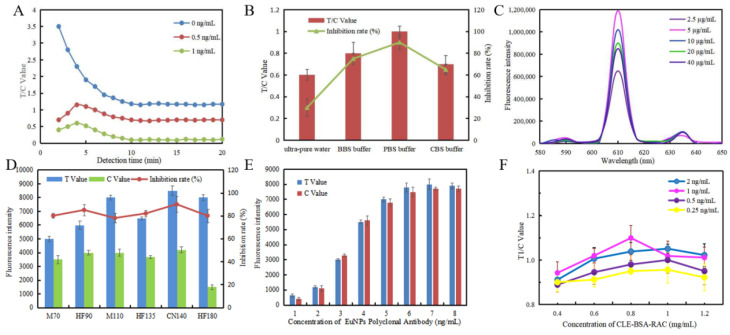
Optimization of the EuNP-FLFIA assay. (**A**) The relationship between detection time and the T/C value. The sample concentration was 0, 0.5, and 1 ng/mL, respectively. (**B**) Effect of buffers on the T/C value. Buffer types: ultra-pure water, BBS buffer, PBS buffer, and CBS buffer. (**C**) Effect of different EuNP polyclonal antibody probe concentrations on the fluorescence intensity of the mixed solutions. (**D**) The fluorescence intensity values and inhibition intensity are shown by using different nitrocellulose membranes. Nitrocellulose membrane types: M70, HF90, M110, HF135, CN140, and HF180. (**E**) Effect of EuNP polyclonal antibody probe concentrations on the fluorescence intensity. (**F**) Influence of various CLE-BSA-RAC concentrations and EuNP polyclonal antibody probe concentrations on the T/C value.

Figure 2A shows the fluorescence response for different reaction times. After each reaction, the T and C values were recorded every 1 min for 20 min. The T/C value showed a sharp change trend with the increase in reaction time in the first 5 min, then stabilized and reached equilibrium within 10 min. It could be seen that the entire reaction time needs to last for 10 min.

The buffer would also have a great impact on the performance of EuNP-FLFIA. Therefore, we performed four kinds of buffers: BBS buffer (0.05 M, pH 8.2), ultra-pure water (pH 5.0), PBS buffer (0.05 M, pH 7.4), and CBS buffer (0.05 M, pH 9.6). The result is shown in Figure 2B. By comparing the T/C value and the inhibition rate, the PBS buffer was considered to be the best buffer.

As shown in Figure 2C, the fluorescence intensity was highest when the amount of antibody labeled was 5 µg/mL. It did not increase with the increase in antibody concentration. Therefore, the optimal concentration of the polyclonal antibody was 5 µg/mL.

The nitrocellulose (NC) membrane also had a significant impact on the results of FICTS. We tested six types of NC membranes, namely MDI 70CNPH-N-SS40 (M70), Millipore 90 (HF90), MDI CNPF-SN12 (M110), Millipore 135 (HF135), Sartorius CN140 (CN140), and Millipore 180 (HF180). The result is shown in Figure 2D. By comparing the fluorescence intensity and the inhibition intensity, it could be seen that the Sartorius CN140-type NC membrane was the best choice for low background noise, high sensitivity, and high fluorescence signal intensity.

As shown in Figure 2E, the fluorescence intensity increased with the increase in the concentration of EuNP polyclonal antibodies, and the fluorescence intensity value tended to be stable at a concentration of 6 ng/mL.

To achieve the best analysis performance for the EuNP-FLFIA, the concentration of coated CLE-BSA-RAC and goat anti-rabbit IgG were optimized through cross-reaction. The results are shown in Figure 2F. The amount of coated antigen was 0.4, 0.6, 0.8, 1.0, and 1.2 mg/mL, and the goat anti-rabbit IgG was 0.25, 0.5, 1, and 2 ng/mL, respectively. According to the results, considering the minimum actual dosage and the best display signal, the optimal concentration combination of CLE-BSA-RAC on the T line and EuNP polyclonal antibody probes concentration was: the optimal dosage of CLE-BSA-RAC was 0.8 mg/mL, and the goat anti-rabbit IgG concentration was 1 ng/mL.

### 3.4. Sensitivity and Specificity Determination

**Figure 3:**
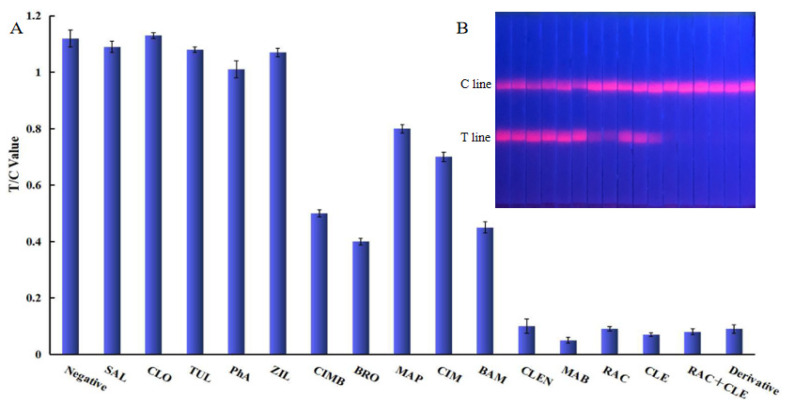
Specific analysis of EuNP-FLFIA. (**A**) Specific evaluation results (from left to right are negative: SAL, CLO, TUL, PhA, ZIL, CIMB, BRO, MAP, CIM, BAM, CLEN, MAB, RAC, CLE, RAC+CLE, and Derivative). (**B**) T/C value data analysis showing the extent of the cross-reactivity of CLE-RAC with other *β*_2_-agonists.

The specificity of the established EuNP-FLFIA was evaluated with 14 commonly used *β*_2_-agonists. As shown in Figure 3, the results showed that the obtained polyclonal antibody exhibited high cross-reactivity with eight aniline-types and one phenol-type *β*_2_-agonist, while its cross-reactivity (CR) with other *β*_2_-agonists was negligible. The cross-reactivity of MAB was found to be high in this study. An analysis of the reason for this showed that the structures of MAB and CLE were very similar, so the cross-reaction rate was also high (Supplementary Materials Table S2).

**Figure 4:**
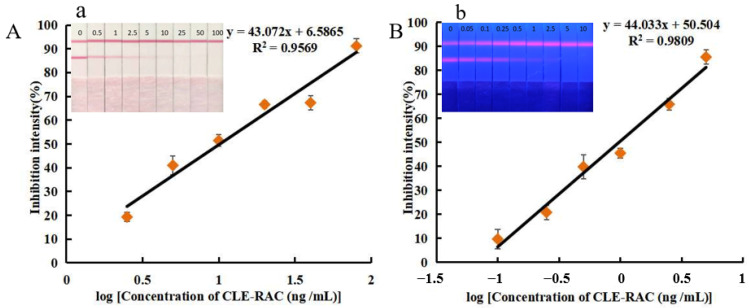
Detection in EuNP-FLFIA and CG-LFIA. (**A**). The standard curve for CLE+RAC (1:1) by CG-LFIA. a. Physical diagram of CG-LFIA for CLE-RAC sensitivity detection. (**B**). The standard curve for CLE+RAC (1:1) by EuNP-LFIA. b. Physical diagram of EuNP-FLFIA for CLE-RAC sensitivity detection.

By optimizing the experimental conditions, the competitive form-based detection methods for CG-LFIA and EuNP-FLFIA were established. A total of eight aniline-type and one phenol-type *β*_2_-agonist with specificity were tested, and the sensitivity of the test strip was observed by increasing the standard concentration. For testing using the EuNP-FLFIA method, a standard curve was created by reading the loaded strip using a fluorescence reader, as shown in Figure 4B. The CG-LFIA method was similar to that described above, as shown in Figure 4A. The single *β*_2_-agonist standard curves are shown in Figure S4. Therefore, as shown in Table 1, the IC_50_ value of the anilino-type *β*_2_-agonists as measured using the CG-LFIA method was 9.92–29.83 ng/mL, and the LOD was 1.14–4.08 ng/mL. The IC_50_ value for the phenol-type was 10.42 ng/mL, and the LOD was 1.12 ng/mL. The IC_50_ value of the anilino-type *β*_2_-agonists was 0.89 to 2.86 ng/mL, and the LOD was 0.11–0.19 ng/mL using the EuNP-FLFIA method; the IC_50_ value for the phenol-type was 0.98 ng/mL, and the LOD was 0.12 ng/mL. The significantly different sensitivities of CG-LFIA and EuNP-FLFIA led to the conclusion that EuNPs were more suitable probes for this study than the controls. This method achieved the level of detection of a single *β*_2_-agonist with improved sensitivity compared to the other methods (Supplementary Materials Table S3).

### 3.5. Detection of Spiked Samples by EuNP-FLFIA

In this experiment, this method was used to detect *β*_2_-agonist-negative swine urine samples added with different concentrations. The method’s accuracy was evaluated by calculating the ratio of the actually detected concentration to the added concentration. Additionally, we repeated the experiment three times for each concentration. The accuracy and repeatability between batches were reflected in the relative standard deviation (RSD). The test results are shown in Table 2. The recovery rate of the spiked samples detected by this method was 84.00%–114.00%. The RSD was less than 8.01%. The results showed that this method could better distinguish spiked samples, had high consistency and accuracy, and could be used for the quantitative analysis.

### 3.6. Detection of Actual Samples by EuNP-FLFIA and LC-MS/MS

**Figure 5:**
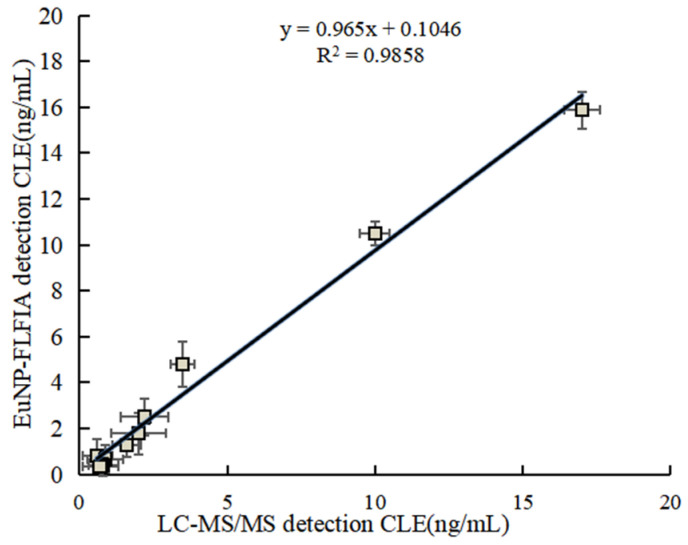
EuNP-FLFIA and LC-MS/MS comparison test results.

To further test and verify the accuracy of the EuNP-FLFIA method, as shown in Table 3, EuNP-FLFIA and LC-MS/MS analyses were used to analyze 60 swine urine, serum, muscle, and liver samples around Zhejiang Province. To ensure the accuracy of the analysis results, each sample was analyzed three times to compare the correlation between the two methods. The results in Figure 5 show satisfactory consistency between the detected values of EuNP-FLFIA and LC-MS/MS (R^2^ > 0.98). The results showed that the correlation coefficient was greater than 0.98, indicating that EuNP-FLFIA has a good correlation with LC-MS/MS and could be reliably used for the detection of *β*_2_-agonists. Therefore, the method could effectively detect the *β*_2_-agonists drug residues in the actual samples and be used for food safety detection.

## 4. Discussion

The abuse of *β*_2_-agonists led to the accumulation of drugs in animals. Eating contaminated animal tissues could cause drugs to enter the human body and thus endanger their health. Therefore, the detection of drug residues in *β*_2_-agonists was particularly important. In general, there are two types of *β*_2_-agonist drug residues in animals. Therefore, detecting two targets simultaneously with one experiment is important to ensure food safety.

The *β*_2_-agonists had a low molecular weight and were not immunogenic. Therefore, they must be coupled with the carrier protein to stimulate the body to produce the corresponding antibody [38]. The synthetic method selected in this experiment was a diazotization reaction, aiming to link CLE and RAC to the same carrier protein. However, since RAC could not directly participate in the diazotization reaction [39], in this experiment, the structure of RAC was redesigned by adding an amino group. RAC amino derivatives were prepared from hydroxybenzaldehyde and nitromethane. RAC-NH_2_ and CLE participated in the diazotization and coupling reactions of the carrier protein together to prepare the dimer artificial antigen to achieve the effect of simultaneously detecting two types of *β*_2_-agonists.

The sensitivity of the immunochromatographic assay techniques depended on the probes formed by antibodies and nanomaterials. The development of a fluorescence immunoassay has attracted extensive attention in recent years [40,41]. In this study, high-quality fluorescent probes were prepared using EuNPs and polyclonal antibodies. The addition of fluorescent probes to the immune test strips significantly increased the sensitivity of the detection of *β*_2_-agonist residues in pig products, with portable fluorescent readers producing results within 10 min.

Table S3 summarizes the comparison between this method and other methods for detecting *β*_2_-agonist residues. The research method established in this paper not only had high detection sensitivity, which could basically achieve the sensitivity of detecting a single analyte [18,27], but could also detect nine phenol-type and aniline-type *β*_2_-agonists simultaneously in a single time, with the detection time reduced to less than 10 min [42,43], which laid a good foundation for future research. To confirm the validity and correctness of FLFIA analysis, this study analyzed the recovery rate of *β*_2_-agonists spiked in pig urine. The results showed that this method had high consistency and accuracy and could be used for quantitative analysis. The LC-MS/MS method was used as a validation method for judging the reliability of the EuNP-FLFIA assay developed in this paper. By detecting actual samples, a good correlation (R^2^ > 0.98) was shown in Figure 5. It meant that the measurements of the EuNP-FLFIA assay were reliable.

The immunoassay method selective for two *β*_2_-agonists of aniline and phenol established in this paper was not only high in sensitivity but also could save costs and time and had an important application value. In the future, the development and establishment of fluorescent immunoassay test strips will ensure the safety of various foods. We will further study and improve this method to detect real samples.

## 5. Conclusions

RAC-NH_2_, an amino derivative of RAC, was de novo synthesized from p-hydroxybenzaldehyde and nitromethane by the Raney nickel reduction method. The product was used as the starting material for diazotization reaction with CLE to synthesize the dimer artificial antigen, which was used to immunize the New Zealand rabbits by the conventional immune method to obtain the polyclonal antibody. Then, the EuNP-FLFIA detection system was constructed by combining the polyclonal antibody, fluorescent material europium nanoparticles, and an immunochromatographic assay. A total of eight aniline-type *β*_2_-agonists and one phenol-type *β*_2_-agonists could be detected quickly and with high sensitivity. The method not only solved the problems of complex operation and low sensitivity in the traditional experiment but also solved the problem that only one type of *β*_2_-agonists could be detected at a time and realized the simultaneous detection of two types of substances of the *β*_2_-agonists. In the detection of actual samples, by comparing the results of LC-MS/MS and EuNP-FLFIA, the correlation between the two was high (R^2^ > 98%), indicating that the detection method established in this study was reliable. The method had the advantages of high sensitivity, good stability, simple operation, and provided a good tool for food safety detection.

## Figures and Tables

**Table 1:** Standard curve equations for the analysis of *β*_2_-agonists using CG-LFIA and EuNP-FLFIA.

**CG-LFIA**				
***β*_2_-Agonists**	**Standard Curve**	**R^2^**	**IC_50_** **(ng/mL)**	**LOD** **(ng/mL)**
CLE	y = 42.123x + 7.6785	0.96	10.11	1.14
RAC	y = 41.364x + 7.8854	0.95	10.42	1.12
MAB	y = 43.167x + 6.9773	0.95	9.92	1.17
BAM	y = 45.243x − 4.1525	0.95	15.74	2.04
BRO	y = 49.039x − 11.854	0.97	16.94	2.75
MAP	y = 46.332x − 18.323	0.96	29.83	4.08
CIM	y = 46.332x − 15.323	0.96	25.70	2.14
CLEN	y = 44.707x + 3.6362	0.95	10.89	1.38
CIMB	y = 48.816x − 17.775	0.97	24.43	3.72
CLE+RAC	y = 43.072x + 6.5865	0.96	10.19	1.20
Mixed solution	y = 41.921x + 3.8361	0.96	12.59	1.40
**EuNP-FLFIA**				
***β*_2_-Agonists**	**Standard Curve**	**R^2^**	**IC** **_50_** **(ng/mL)**	**LOD** **(ng/mL)**
CLE	y = 43.349x + 50.829	0.98	0.96	0.11
RAC	y = 44.468x + 50.396	0.98	0.98	0.12
MAB	y = 43.966x + 52.329	0.97	0.89	0.11
BAM	y = 39.138x + 44.763	0.97	1.36	0.13
BRO	y = 36.708x + 42.77	0.97	1.58	0.13
MAP	y = 34.149x + 34.426	0.98	2.86	0.19
CIM	y = 33.415x + 35.994	0.97	2.62	0.17
CLEN	y = 42.33x + 49.775	0.97	1.01	0.11
CIMB	y = 34.463x + 41.135	0.97	1.80	0.13
CLE+RAC	y = 44.033x + 50.504	0.98	0.97	0.12
Mixed solution	y = 37.379x + 46.526	0.97	1.24	0.11

**Table 2:** Recovery of *β*_2_-agonists in swine urine samples by EuNP-FLFIA.

Target	Spiked Level (ng/mL)	Intra-Assay ^a^			Target	Spiked Level (ng/mL)	Intra-Assay ^a^		
		Detected Amount (ng/mL)	Recovery Rate	RSD (*n* = 3)			Detected Amount (ng/mL)	Recovery Rate	RSD (*n* = 3)
Clenbuterol	0.5	0.44	88.00%	6.53%	Ractopamine	0.5	0.57	114.00%	6.05%
	1.0	1.09	109.00%	4.56%		1.0	1.06	106.00%	2.28%
	2.0	2.18	109.00%	8.01%		2.0	2.21	110.50%	4.49%
Mabuterol	0.5	0.42	84.00%	5.56%	Bambuterol	0.5	0.49	98.00%	5.86%
	1.0	0.85	85.00%	3.59%		1.0	0.87	87.00%	4.92%
	2.0	1.96	98.00%	3.37%		2.0	1.73	86.50%	2.37%
Brombuterol	0.5	0.52	104.00%	5.83%	Mapenterol	0.5	0.47	94.00%	4.60%
	1.0	1.14	114.00%	6.43%		1.0	0.94	94.00%	4.52%
	2.0	2.16	108.00%	5.81%		2.0	1.77	88.50%	4.20%
Cimaterol	0.5	0.55	110.00%	6.83%	Clenproperol	0.5	0.47	94.00%	6.77%
	1.0	1.12	112.00%	5.82%		1.0	0.91	91.00%	4.60%
	2.0	1.83	91.50%	2.80%		2.0	2.09	104.50%	1.98%
Cimbuterol	0.5	0.48	96.00%	5.82%	CLE + RAC	0.5	0.55	110.00%	5.85%
	1.0	0.88	88.00%	1.12%	(1:1)	1.0	1.02	102.00%	4.78%
	2.0	1.99	99.50%	1.45%		2.0	2.12	106.00%	2.64%

^a^ Repeat assay (*n* = 3).

**Table 3:** Detection of *β*_2_-agonists in actual samples from EuNP-FLFIA with LC-MS/MS.

Sample	EuNP-FLFIA ^a^ (ng/mL)	LC-MS/MS ^a^ (ng/mL)	Sample	EuNP-FLFIA ^a^ (ng/mL)	LC-MS/MS ^a^ (ng/mL)
Urine 01	/ ^b^	/	Urine 11	/	/
Urine 02	/	/	Urine 12	1.78 ± 0.09	1.93 ± 0.12
Urine 03	/	/	Urine 13	/	/
Urine 04	/	/	Urine 14	/	/
Urine 05	/	/	Urine 15	/	/
Urine 06	0.81 ± 0.22	0.80 ± 0.09	Urine 16	/	/
Urine 07	/	/	Urine 17	/	/
Urine 08	/	/	Urine 18	/	/
Urine 09	0.67 ± 0.13	0.88 ± 0.15	Urine 19	/	/
Urine 10	/	/	Urine 20	0.33 ± 0.04	0.36 ± 0.02
Muscle 01	/	/	Muscle 10	/	/
Muscle 02	/	/	Muscle 11	/	/
Muscle 03	/	/	Muscle 12	/	/
Muscle 04	/	/	Muscle 13	0.41 ± 0.23	0.45 ± 0.16
Muscle 05	/	/	Muscle 14	/	/
Muscle 06	/	/	Muscle 15	/	/
Muscle 07	/	/	Muscle 16	/	/
Muscle 08	1.27 ± 0.14	1.38 ± 0.11	Muscle 17	/	/
Muscle 09	/	/	Muscle 18	/	/
Liver 01	/	/	Liver 07	/	/
Liver 02	10.22 ± 0.31	10.31 ± 0.20	Liver 08	/	/
Liver 03	/	/	Liver 09	/	/
Liver 04	/	/	Liver 10	15.88 ± 0.24	16.02 ± 0.14
Liver 05	/	/	Liver 11	/	/
Liver 06	/	/	Liver 12	/	/
Serum 01	/	/	Serum 06	2.11 ± 0.11	2.13 ± 0.03
Serum 02	/	/	Serum 07	/	/
Serum 03	4.21 ± 0.08	4.27 ± 0.05	Serum 08	/	/
Serum 04	/	/	Serum 09	/	/
Serum 05	/	/	Serum 10	/	/

^a^ Repeat assay (*n* = 3). ^b^ Not detected.

## Data Availability

The original contributions presented in the study are included in the article/Supplementary Materials, further inquiries can be directed to the corresponding author.

## Supplementary Materials

The following are available online at https://www.mdpi.com/article/10.3390/foods11060863/s1: Figure S1. Schematic diagram of the synthesis of dimerized artificial antigens. Figure S2. UV spectrum of the CLE-RAC artificial antigens. (a) UV spectrum scan of CIT–BSA. (From left to right, the molar ratio of the small molecule to carrier protein was 100, 150, and 200, respectively). (b) UV spectrum scan of CIT-HSA. (From left to right, the molar ratio of the small molecule to carrier protein was 100, 150, and 200, respectively). Figure S3. SDS-PAGE electrophoresis of CLE-RAC artificial antigens. (a) SDS-PAGE electropherogram of CLE-BSA-RAC. (b) SDS-PAGE electropherogram of CLE-HSA-RAC. Figure S4. Detection in EuNP-FLFIA and CG-LFIA. (a–j) CLE, RAC, MAB, BAM, BRO, MAP, CIM, CLENP, CIMB, and mixed solution, respectively). Table S1.Titer of antiserum detected by indirect ELISA. Table S2. Cross-reaction of CLE-RAC polyclonal antibody with *β*_2_-agonists and antibiotic. Table S3. Comparison of the analytical performance of different detection probes for the detection of *β*_2_-agonist residues.
