# Supplementary material for: Research on Rapid Detection Technology for β2-Agonists: Multi-Residue Fluorescence Immunochromatography Based on Dimeric Artificial Antigen
Source: Foods. 2022 Mar 18;11(6):863. doi: 10.3390/foods11060863 (PMC8949518; doi:10.3390/foods11060863)
Supplement: Supplementary file 1 [file foods-11-00863-s001.zip › foods-1596338-supplementary.pdf]

Supplementary Materials:

Research on rapid detection technology for β_2-agonists: multi-residue fluorescence immunochromatography based on dimeric artificial antigen

S1. Synthesis of CLE-RAC Artificial Antigen

The RAC-NH_2 derivative and CLE were mixed in equal proportions and participated in the diazotization reaction as a small molecular substance. The molar ratio of adding the two small molecules together to the protein selects three ratios: 100, 150, and 200, and we selected a dimeric artificial antigen with the best synthesis effect for subsequent experiments.

A schematic diagram of the synthesis of dimeric artificial antigens is shown in Figure S1.

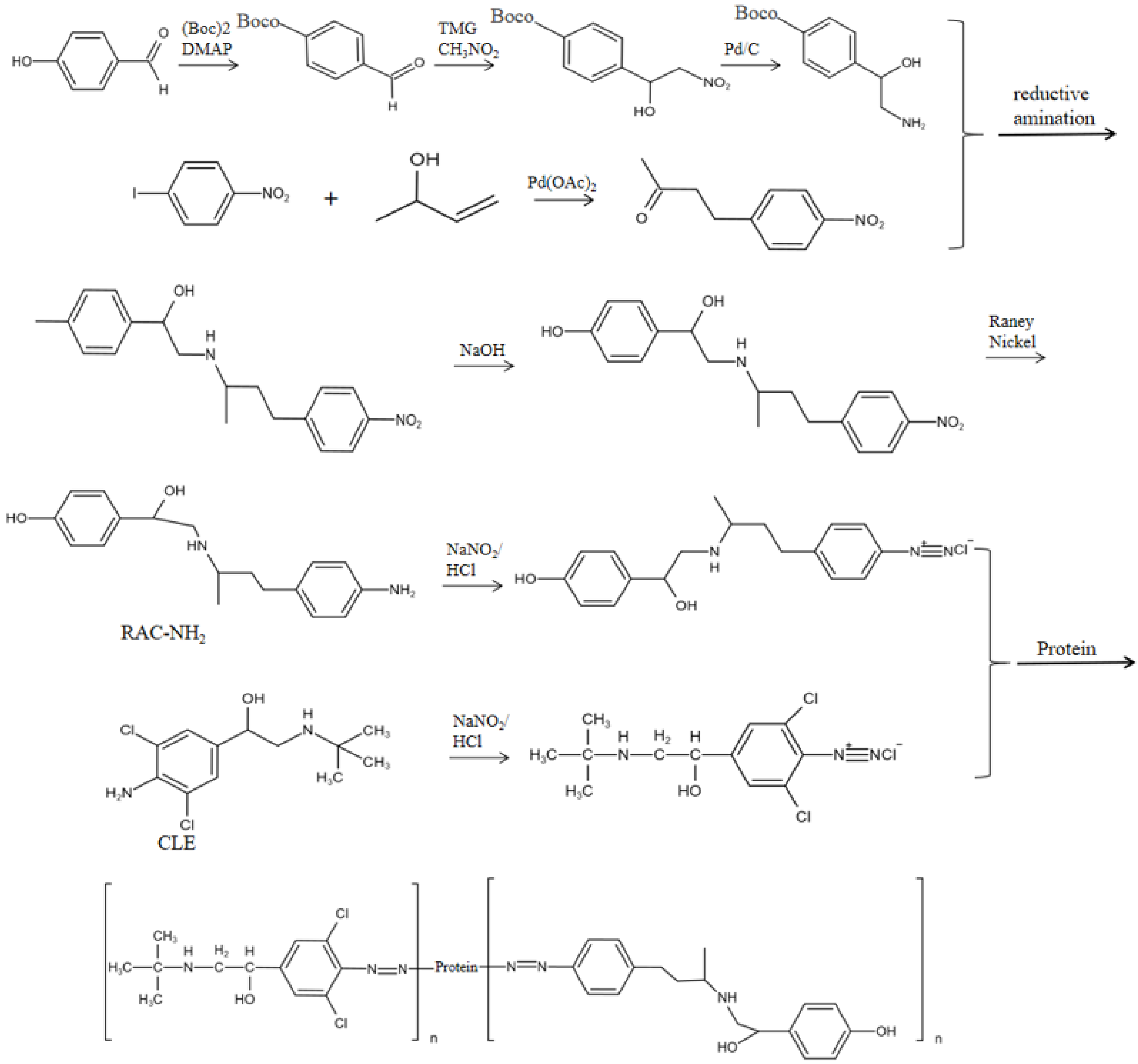

Figure S1. Schematic diagram of the synthesis of dimerized artificial antigens.

S2. Identification of the CLE-RAC artificial antigen

Clenbuterol (CLE) and Ractopamine (RAC) are non-immunogenic, small molecular weight β_2-agonists. Therefore, they are coupled to a carrier protein to obtain a specific polyclonal antibody. To test for successful coupling, BSA, HSA, CLE-BSA-RAC, and CLE-HSA-RAC were tested by UV-Vis spectroscopy and 12% SDS-PAGE, as shown in Figures S2 and S3.

It could be seen from Figure S2 that the characteristic absorption peak of CLE was at 294 nm, the characteristic absorption peak of BSA was at 278 nm, the characteristic absorption peak of RAC was at 275 nm, and the characteristic absorption peak of HSA was at 279 nm. The conjugate formed by the coupling reaction with BSA had two characteristic absorption peaks at 279 nm and 331 nm; the conjugate that underwent the coupling reaction with HSA had two characteristic absorption peaks at 281 nm and 332 nm. It could be seen from the figure that when the molar ratio of the small molecule to protein was 100, the peak value of the characteristic absorption peak was the highest and obvious, which indicated that the coupling reaction with the carrier protein in the experiment might be successful.

Next was the SDS-PAGE electrophoresis assay. As shown in Figure S3a, the CLE-BSA-RAC band significantly lagged behind that of BSA, so CLE and RAC may successfully bind to the residues of BSA to form conjugations. Similarly, CLE and RAC may also successfully couple to HSA (Figure S3b). Therefore, the dimeric artificial antigen with a molar ratio of 100 was selected for subsequent experiments.

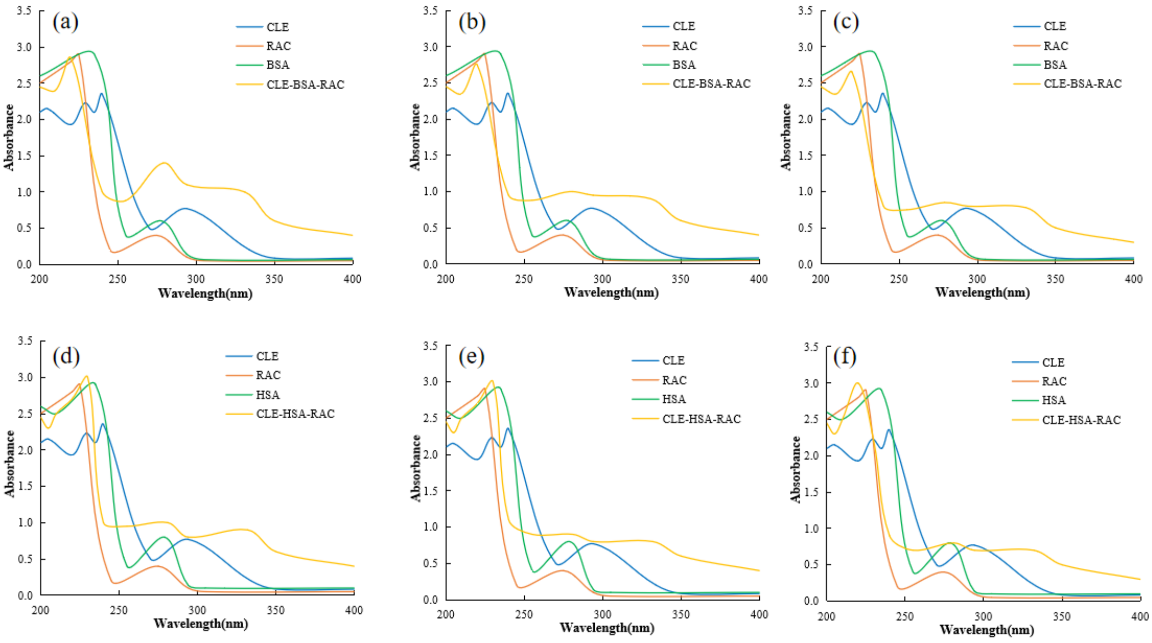

Figure S2. UV spectrum of the CLE-RAC artificial antigens. (a–c): UV spectrum scan of CLE-BSA-RAC (from left to right, the molar ratio of CLE and RAC-NH_2 together to carrier protein was 100, 150, and 200, respectively). (d–f): UV spectrum scan of CLE-HAS-RAC (from left to right, the molar ratio of CLE and RAC-NH_2 together to carrier protein was 100, 150, and 200, respectively).

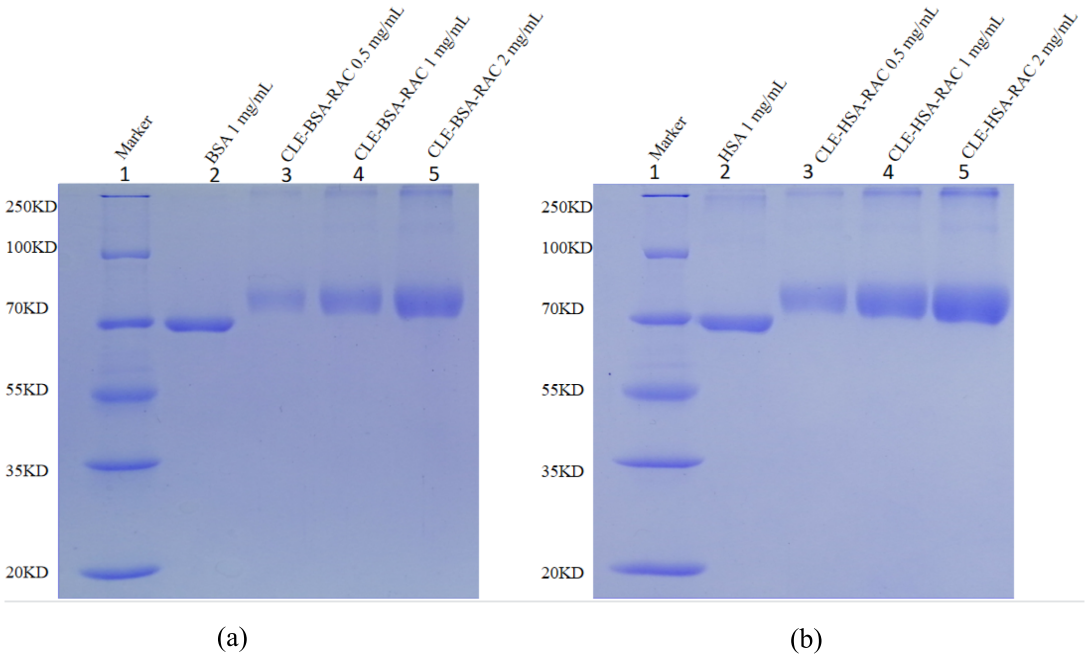

Figure S3. SDS-PAGE electrophoresis of CLE-RAC artificial antigens. (**a**) SDS-PAGE electropherogram of CLE-BSA-RAC. (**b**) SDS-PAGE electropherogram of CLE-HSA-RAC.

S3. Identification of the anti-CLE-RAC polyclonal antibody

The coated original CLE-BSA-RAC was diluted 1: 10000 times with the coating solution, and 96-well polystyrene plates were coated with 50 μL per well. The plates were incubated for 2 h at 37 ℃ and washed four times with the washing solution (the same below). The wells were blocked with 250 μL of blocking solution per well, incubated for 1 h at 37 ℃, and washed. They were air-dried then put in a refrigerator at 4 ℃ for later use. Polystyrene plates coated with CLE-BSA-RAC were taken and each well was bedded with 50 μL diluent. The first well had 50 μL of antiserum added, diluted 1: 4,000 times and then diluted in a times ratio. The negative control (NC) and blank control (BC) were set and incubated at 37 ℃ for 15 min, followed by plate washing. The samples had IgG-HRP added, diluted with dilute release solution at a ratio of 1: 5 000, 50 μL per well, were incubated at 37 ℃ for 30 min, and plated. Then, the color solution (50 μL per well) was added for reaction at room temperature for 5 min. After the color reaction was stopped, the stop solution was added at 50 μL per well, and the OD_{450} value was read by an ELISA reader. As shown in Table S1, the antiserum titers of the two immunized New Zealand rabbits reached more than 1: 128000, indicating that the injected immunogen achieved a good immune effect.

Table S1. Titer of antiserum detected by indirect ELISA

Number	Dilution multiple							Negative	Blank
	4000	8000	16000	32000	64000	128000	256000		
1	2.482	2.443	1.284	0.835	0.621	0.357	0.135	0.089	0.077
2	3.211	2.621	1.389	0.779	0.698	0.324	0.123	0.083	0.075

S4. Sensitivity and specificity determination

To identify the specificity of polyclonal antibodies, this experiment detected the IC_{50} values of CLE, RAC, phenylethanolamine A, Salbutamol, Cloprenaline, Cimbuterol,

Bromobuterol, Tulobuterol, Mapenterol, Cimaterol, Mabuterol, Zilpaterol, Bambuterol, and Clenproperol. The specificity of the antibody was evaluated by calculating the cross-reaction rate (CR) between these competing substances and the CLE-RAC polyclonal antibody. The results are presented in Table S2. In this study, it was found that the CR of the CLE-RAC polyclonal antibody and MAB was 107.87%. It had a higher rate of cross-reaction with CLE and RAC. Through observation, it was found that the structural formula of Mabuterol was very similar to that of CLE. Probably because of this reason, the CR of CLE-RAC polyclonal antibody to it is so high. The other several possibilities were also due to the similarity of the structure, which will have a higher CR. Therefore, the detection method established in this study can specifically identify the multi-residue stimulant and realize multi-residue detection.

The single β_2-agonists standard curves are shown in Figure S4.

Table S2. Cross-reaction of CLE-RAC polyclonal antibody with β_2-agonists and antibiotic

Compounds	IC_{50} (ng/mL)	Cross-reactivity (%)
Clenbuterol	0.96	100.00
Ractopamine	0.98	97.96
Salbutamol	>810	<0.10
Phenylethanolamine A	>810	<0.10
Clorprenaline	>810	<0.10
Cimbuterol	1.80	53.33
Brombuterol	1.58	60.76
Tulobuterol	>810	<0.10
Mapenterol	2.86	33.57
Cimaterol	2.62	36.54
Mabuterol	0.89	107.87
Zilpaterol	>810	<0.10
Bambuterol	1.36	70.59
Clenproperol	1.01	95.05

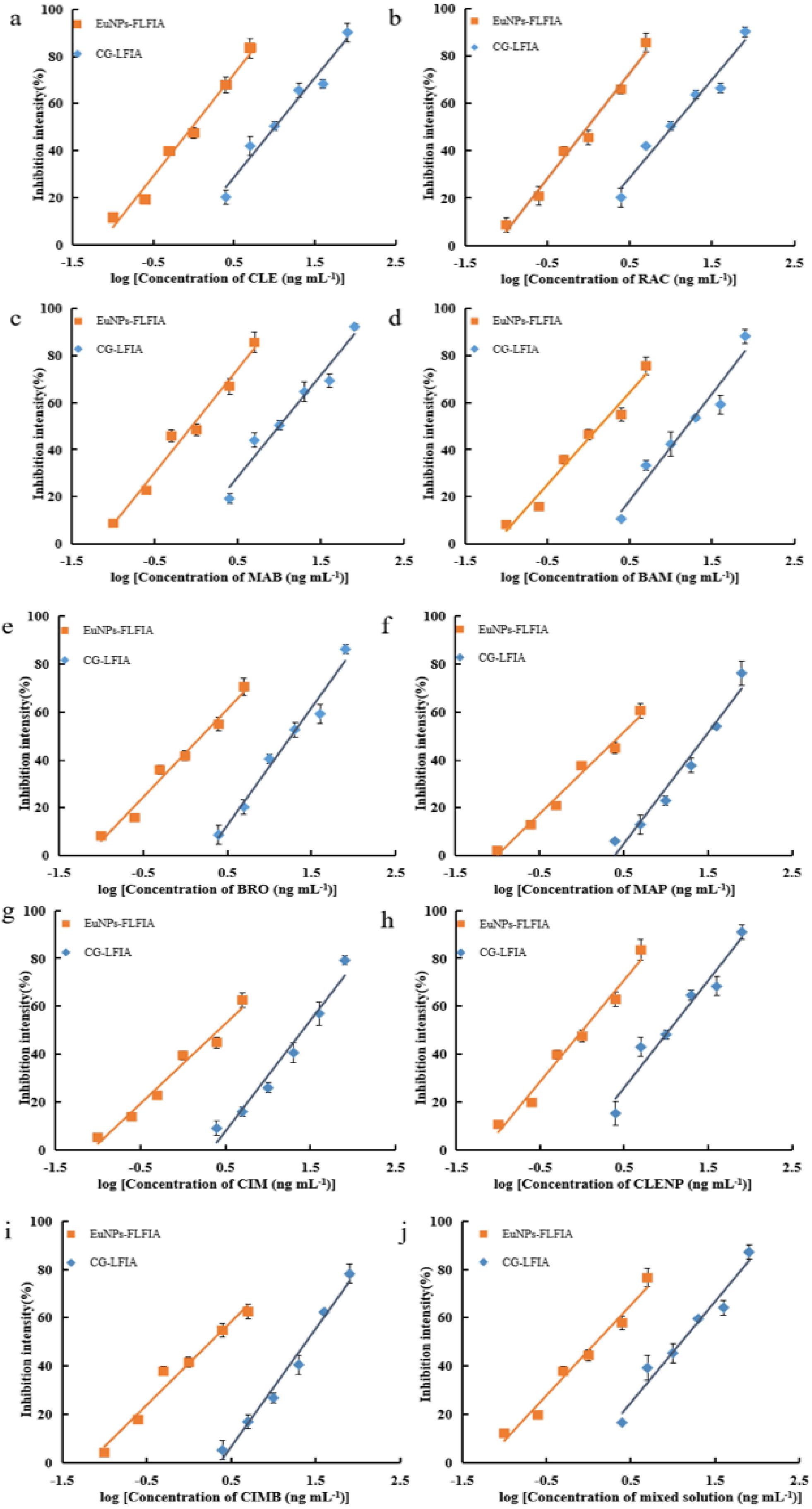
a
EuNPs-FLFIA
CG-LFIA
Inhibition intensity(%)
log [Concentration of CLE (ng mL-1)]
b
EuNPs-FLFIA
CG-LFIA
Inhibition intensity(%)
log [Concentration of RAC (ng mL-1)]
c
EuNPs-FLFIA
CG-LFIA
Inhibition intensity(%)
log [Concentration of MAB (ng mL-1)]
d
EuNPs-FLFIA
CG-LFIA
Inhibition intensity(%)
log [Concentration of BAM (ng mL-1)]
e
EuNPs-FLFIA
CG-LFIA
Inhibition intensity(%)
log [Concentration of BRO (ng mL-1)]
f
EuNPs-FLFIA
CG-LFIA
Inhibition intensity(%)
log [Concentration of MAP (ng mL-1)]
g
EuNPs-FLFIA
CG-LFIA
Inhibition intensity(%)
log [Concentration of CIM (ng mL-1)]
h
EuNPs-FLFIA
CG-LFIA
Inhibition intensity(%)
log [Concentration of CLENP (ng mL-1)]
i
EuNPs-FLFIA
CG-LFIA
Inhibition intensity(%)
log [Concentration of CIMB (ng mL-1)]
j
EuNPs-FLFIA
CG-LFIA
Inhibition intensity(%)
log [Concentration of mixed solution (ng mL-1)]

Figure S4. Detection in EuNP-FLFIA and CG-LFIA. (a–j are CLE, RAC, MAB, BAM, BRO, MAP, CIM, CLENP, CIMB and mixed solution, respectively).

S5. Comparison of different methods for the detection of *β*2-agonist residues

Table S3. Comparison of the analytical performance of different detection probes for the detection of *β*2-agonist residues.

Detection Method	Analytes	Sample	Detection time (min)	LOD	References
Se-NPs	ractopamine<br>salbutamol	swine urine	5	1 ng/mL<br>3 ng/mL	[1]
MLFIA	ractopamine<br>clenbuterol	pork and mutton	-	6 ng/mL<br>12 ng/mL	[2]
SiNPs	clenbuterol	-	-	1.6 ng/mL	[3]
AuNPs	clenbuterol	-	20	2 ng/mL	[4]
AuNPs	salbutamol<br>clenbuterol<br>cimaterol,<br>brombuterol<br>mabutero<br>terbutaline,<br>clenpenterol,<br>carbuterol,<br>mapenterol<br>pirbuterol	swine urine	20	5 ng/mL<br>5 ng/mL<br>20 ng/mL<br>20 ng/mL<br>20 ng/mL<br>50 ng/mL<br>50 ng/mL<br>50 ng/mL<br>50 ng/mL<br>50 ng/mL	[5]
EuNP-FLFIA	clenbuterol<br>ractopamine<br>cimbuterol<br>brombuterol<br>mapenterol<br>cimaterol<br>mabuterol<br>bambuterol<br>clenproperol	swine urine	10	0.11 ng/mL<br>0.12 ng/mL<br>0.13 ng/mL<br>0.13 ng/mL<br>0.19 ng/mL<br>0.17 ng/mL<br>0.11 ng/mL<br>0.13 ng/mL<br>0.11 ng/mL	This study

Se-NPs: selenium nanoparticles.

MLFIA: a magnetic Prussian blue nanozyme (MPBN) mediated dual-readout on-demand multiplex lateral flow immunoassay.

SiNPs: porous silica nanoparticles.

AuNPs: gold nanoparticles.

EuNP-FLFIA: europium nanoparticle-based fluorescent immunoassay.

The limit of detection (LOD) was calculated as the mean of the measured content of different blank samples (n=20) plus three standard deviations (mean+3SD). We have calculated the LODs of swine urine according to this calculation method were 0.6 ng/mL, 0.6

ng/mL, 2 ng/mL, 1 ng/mL, 3 ng/mL, 3 ng/mL, 1 ng/mL, 1 ng/mL, and 1ng/mL, respectively.

References

1. Wang, Z.Z.; Zhou, Q.Q.; Guo, Y.F.; Hu, H.Z.; Zheng, Z.; Li, S.L.; Wang, Y.H.; Ma, Y.F. Rapid Detection of Ractopamine and Salbutamol in Swine Urine by Immunochromatography Based on Selenium Nanoparticles. *Int J Nanomedicine*, **2021**, *16*, 2059-2070. Doi:10.2147/IJN.S292648.
2. Liu, S.J.; Dou, L.N.; Yao, X.L.; Zhang, W.T.; Zhao, M.; Yin, X.C.; Sun, J.; Zhang, D.H.; Wang, J.L. Nanozyme amplification mediated on-demand multiplex lateral flow immunoassay with dual-readout and broadened detection range. *Biosens Bioelectron*, **2020**, *169*, 112610. DOI:10.1016/j.bios.2020.112610.
3. Song, C.M.; Zhi, A.M.; Liu, Q.T.; Yang, J.F.; Jia, G.C.; Shervin, J.; Tang, L.; Hu, X.F.; Deng, R.G.; Xu, C.L.; Zhang, G.P. Rapid and sensitive detection of β-agonists using a portable fluorescence biosensor based on fluorescent nanosilica and a lateral flow test strip. *Biosens Bioelectron*, **2013**, *50*, 62-5. DOI: 10.1016/j.bios.2013.06.022.
4. Huang, Q.; Bu, T.; Zhang, W.T.; Yan, L.Z.; Zhang, M.Y.; Yang, Q.F.; Huang, L.J.; Yang, B.W.; Hu, N.; Suo, Y.R.; Wang, J.L.; Zhang, D.H. An improved clenbuterol detection by immunochromatographic assay with bacteria@Au composite as signal amplifier. *Food Chem*, **2018**, *262*, 48-55. DOI:10.1016/j.foodchem.2018.04.085.
5. Liu, R.; Liu, L.Q.; Song, S.S.; Cui, G.; Zheng, Q.K.; Kuang, H.; Xu, C.L. Development of an immunochromatographic strip for the rapid detection of 10 β-agonists based on an ultrasensitive monoclonal antibody. *Food and Agricultural Immunology*, **2017**, *28*, 625-638. DOI:10.1080/09540105.2017.1309358.
